# Pre-transplant donor specific antibodies in ABO incompatible kidney transplantation – data from the Swiss transplant cohort study

**DOI:** 10.3389/fimmu.2024.1355128

**Published:** 2024-02-01

**Authors:** Yun Deng, Lukas Frischnknecht, Caroline Wehmeier, Olivier de Rougemont, Jean Villard, Sylvie Ferrari-Lacraz, Déla Golshayan, Monique Gannagé, Isabelle Binet, Urs Wirthmueller, Daniel Sidler, Thomas Schachtner, Stefan Schaub, Jakob Nilsson

**Affiliations:** ^1^ Department of Immunology, University Hospital Zurich (USZ), Zurich, Switzerland; ^2^ Clinic for Transplantation Immunology and Nephrology, University Hospital Basel, Basel, Switzerland; ^3^ Department of Surgery and Transplantation, University Hospital Zurich, Zurich, Switzerland; ^4^ Transplantation Immunology Unit and National Reference Laboratory for Histocompatibility, Department of Diagnostic, Geneva University Hospitals, Geneva, Switzerland; ^5^ Transplantation Center, Lausanne University Hospital, Lausanne, Switzerland; ^6^ Service of Immunology and Allergy, Lausanne University Hospital, University of Lausanne, Lausanne, Switzerland; ^7^ Nephrology & Transplantation Medicine, Cantonal Hospital St. Gallen, St. Gallen, Switzerland; ^8^ Department of Laboratory Medicine, Inselspital, Bern University Hospital and University of Bern, Bern, Switzerland; ^9^ Department of Nephrology and Hypertension, Inselspital, Berne University Hospital and University of Berne, Berne, Switzerland; ^10^ Division of Nephrology, University Hospital Zurich, Zurich, Switzerland

**Keywords:** kidney transplantation, ABO incompatible, donor specific antibodies, ABMR, graft loss, virtual cross-match

## Abstract

**Background:**

Living donor (LD) kidney transplantation in the setting of ABO blood group incompatibility (ABOi) has been previously reported to be associated with increased risk for antibody-mediated rejection (ABMR). It is however unclear if the presence of pre-transplant donor specific antibodies (DSA) works as an additive risk factor in the setting of ABOi and if DSA positive ABOi transplants have a significantly worse long-term outcome as compared with ABO compatible (ABOc) DSA positive transplants.

**Methods:**

We investigated the effect of pre-transplant DSA in the ABOi and ABOc setting on the risk of antibody-mediated rejection (ABMR) and graft loss in a cohort of 952 LD kidney transplants.

**Results:**

We found a higher incidence of ABMR in ABOi transplants as compared to ABOc transplants but this did not significantly affect graft survival or overall survival which was similar in both groups. The presence of pre-transplant DSA was associated with a significantly increased risk of ABMR and graft loss both in the ABOi and ABOc setting. We could not detect an additional risk of DSA in the ABOi setting and outcomes were comparable between DSA positive ABOi and ABOc recipients. Furthermore, a combination of DSA directed at both Class I and Class II, as well as DSA with a high mean fluorescence intensity (MFI) showed the strongest relation to ABMR development and graft loss.

**Conclusion:**

The presence of pre-transplant DSA was associated with a significantly worse long-term outcome in both ABOi and ABOc LD kidney transplants and our results suggests that the risk associated with pre-transplant DSA is perhaps not augmented in the ABOi setting. Our study is the first to investigate the long-term effects of DSA in the ABOi setting and argues that pre-transplant DSA risk could potentially be evaluated similarly regardless of ABO compatibility status.

## Introduction

Living donor (LD) kidney transplantation performed in the setting of ABO blood group incompatibility was pioneered over 50 years ago where a complex protocol of repeated plasmapheresis, splenectomy, donor thrombocyte transfusion as well as intensified immunosuppression and infusion of A or B trisaccharide was used (1, 2). The procedure has since then developed significantly and many centers now show comparable outcomes between ABO compatible (ABOc) and ABO incompatible (ABOi) transplantations (3, 4). A protocol for the selective adsorption of anti-ABO antibodies as well as use of Rituximab for B cell depletion was presented by Tydén et al. where extended observation times showed impressive graft survival as well as overall patient survival (5, 6). Variations of this protocol have been adopted by many transplant centers and data from the Collaborative Transplant Study have also indicated that the addition of a B cell depleting therapy (rituximab) is associated with superior outcome (7). Some studies have also shown an inferior overall survival in ABOi transplants as compared to ABOc transplants mainly associated with an increased risk for severe infection in the setting of intensified immunosuppression (8, 9). Despite the more intensive immunosuppression associated with ABOi several studies have also demonstrated an increased risk of antibody mediated rejection (ABMR) in ABOi as compared to ABOc transplants (9, 10). This may in part be due to the universal presence of C4d in ABOi transplants, which makes a diagnosis of ABMR more likely as the Banff Classification does not have a separate algorithm for ABOi transplant biopsies (11). The most significant pre-transplant risk factor for the development of AMBR is the presence of donor specific antibodies (DSA) that target the non-self HLA protein variants in the donor graft (12). DNA based donor HLA typing coupled to regular measurements of anti-HLA antibodies in a recipients serum facilitates the detection of pre-transplant DSA (13). Several previous studies have clearly shown an increased risk of ABMR and graft loss in DSA positive transplantations (12, 14–18). Coupled to the previously described increased risk of ABMR in the ABOi setting this has led to a reluctance in many centers of performing ABOi transplants in the setting of pre-transplant DSA by reasoning that they are additive risk factors that will result in a high risk for ABMR development and graft loss. Previous studies have for the most part not shown an increased risk of graft loss in ABOi patients with pre-transplant DSA as compared to ABOc DSA positive patients, but they have not looked at long-term outcomes and have not been performed in a setting where the virtual cross-match is complete (19–21). In order to improve the pre-transplant immunological risk assessment in the ABOi setting we studied the incidence of ABMR as well as T cell mediated rejection (TCMR), graft loss and overall survival in a cohort of 149 ABOi living donor (LD) kidney transplantations within the Swiss Transplant Cohort Study (STCS) and compared them to 803 living donor ABOc transplants.

## Methods

### Study design and patient population

The Swiss Transplant Cohort Study (STCS, www.stcs.ch) is a multicenter nationwide cohort study conducted in Switzerland. This study (project number FUP142) is a sub-project included within the STCS and separately approved by the Cantonal Ethics Committee of Zurich (BASEC-Nr.2021-0083).

**Figure 1:**
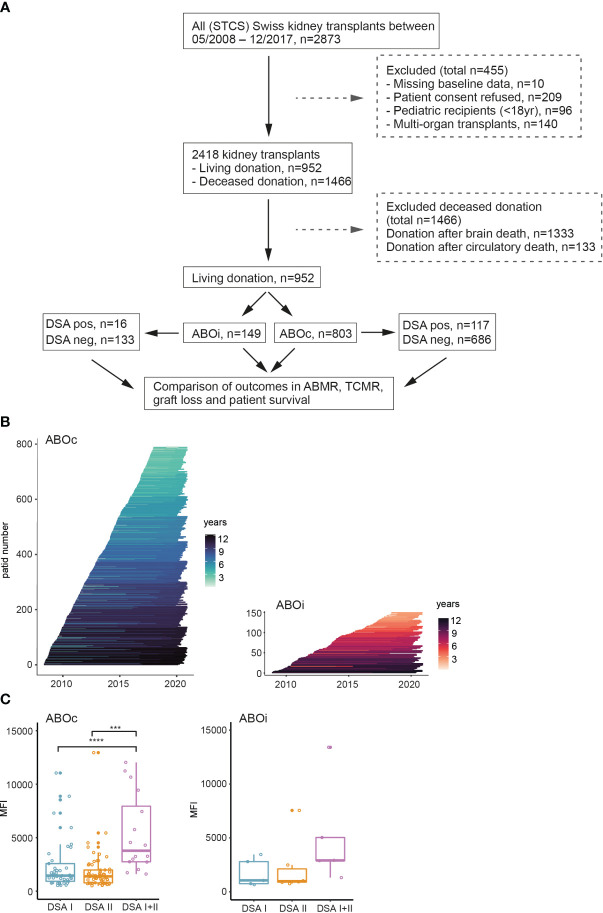
Overview of the ABO study group nested within the STCS cohort. **(A)** Flowchart overview of patient inclusion into the study. **(B)** Follow-up time of each individual patient in the ABOc and ABOi groups. **(C)** Summary of the cumulative DSA MFI in the respective groups. One-way ANOVA analysis with Tukey’s multiple comparisons as a *post hoc* test was used for **(C)** to assess p values; ***p<0.001, ****p<0.0001.

Data from the STCS on kidney transplantations performed between May 2008 and December 2017 (2873 transplantations) were used for this study. In total of 1921 deceased donor transplants were excluded from the analysis, as only patients who received the organ from a LD were included in the current ABO study (n=952). Further exclusion criteria are shown in **Figure 1A**. The outcome analysis was stratified on LD ABOi transplants (n=149) and ABOc transplants (n=803). Detailed information on the included ABOi and ABOc transplants can be seen in **Table 1**.

**Table 1:** Baseline characteristics of the patients.

	ABO-compatible	ABO-incompatible	P-value
N° patients	803	149	
Female gender (Recipient)	272 (33.9%)	41 (27.5%)	0.001
Age (Recipient), mean	49	51	0.175
Female gender (Donor)	507 (63.1%)	95 (63.8%)	0.77
Age (Donor), mean	54	53	0.91
FU (year), mean	6.5	6	0.691
DSA	117	16	0.01
Induction therapy			<0.0001
ATG/Thymo+/- lvlg	141 (17.6%)	11 (7.4%)	
Basiliximab	629 (78.3%)	126 (84.6%)	
None	33 (4.1%)	12 (8.0%)	
ABOi desensitization therapy			
Rituximab based	/	149	
Recipient blood group			<0.0001
A	394 (49.1%)	28 (18.8%)	
B	89 (11.1%)	17 (11.4%)	
O	275 (34.2%)	104 (72.2%)	
AB	45 (5.6%)	/	
Underlying renal disease			0.891
Glomerulonephritis	235 (29.3%)	37 (24.8%)	
ADPKD	155 (19.3%)	39 (26.2%)	
Diabetic nephropathy	54 (6.7%)	7 (4.7%)	
Vascular nephropathy	80 (10.0%)	12 (8.1%)	
Interstitial nephropathy	27 (3.4%)	7 (4.7%)	
Other	19 (2.4%)	1 (0.7%)	
Not specified	89 (11.1%)	21 (14.1%)	
Reflux/Pyelonephritis	48 (6.0%)	10 (6.7%)	
Hereditary (not ADPKD)	32 (4.0%)	3 (2.0%)	
Congenital	23 (2.9%)	6 (4.0%)	
Unknown	41 (5.1%)	6 (4.0%)	

### ABOi treatment protocol in Switzerland

Since 2005 there is a national protocol for LD ABOi kidney transplants in Switzerland (22). A single dose of rituximab (375 mg/m2) was given 4 weeks before the transplantation. Maintenance immunosuppression with tacrolimus (0.1 mg/kg twice daily), mycophenolate mofetil (1000 mg twice daily, 500 mg twice daily if body weight was less than 50 kg), and prednisone (25 mg once daily) was started before transplantation. Selective blood group antibody removal was performed with a low-molecular carbohydrate column containing A or B blood group antigens linked to a sepharose matrix (Glycosorb; Glycorex Transplantation, Lund, Sweden). Apheresis sessions were performed daily until the immunoglobulin (IgG) and isoagglutinin (IgM) antibody titers against donor erythrocytes were 1:8 or less. The transplantation was then carried out the following day. With each session, at least two plasma volumes were processed. At the beginning of the study, a single dose of IVIG (0.5 g/kg body weight) on day −1 was given; later, IVIG therapy was discontinued. The participating Swiss centers were free to choose induction therapy with either basiliximab, ATG or no induction therapy according to the local protocol and based on the individual risk evaluation performed by the treating physicians.

### HLA typing and detection of anti-HLA antibodies

HLA typing was performed on blood samples using either sequence-specific oligonucleotide (SSO) or sequence-specific primer (SSP) technology. Identification of class I and class II HLA antibodies was done using a Luminex bead-based platform (n=948, 99.6%) and ELISA (n=4, 0.4%). While the majority of patients (n=679, 71%) were analyzed with by Luminex single-antigen bead (SAB) technology for the detection of HLA antibodies the rest did not have detectable anti-HLA antibodies based on a mixed bead analysis (LABScreen Mixed, OneLambda) and were thus deemed to be anti-HLA antibody negative. The assessment of the immunologic compatibility between the donor and recipient was done by comparing the donor HLA typing with the recipient anti-HLA antibody profile to generate a virtual crossmatch (vXM). In the event that the recipient had detectable anti-HLA antibodies against a locus that was not previously typed in the donor additional typing was performed to facilitate a complete vXM for all included patients.

### Diagnosis of rejection and definition of graft loss

Graft failure was defined as the initiation of dialysis after transplantation or if preemptive re-transplantation was required. Transplant rejection was defined based on the Banff 2017 criteria (11). The diagnosis of ABMR and TCMR were both biopsy-proven and biopsies were obtained according to the local protocol at each transplant center. The diagnosis of the biopsy was performed by specialized pathologists at each center according to the local protocol. It was documented either as a Banff score or text, which was then translated and graded into individual Banff scores. Biopsies with findings of “borderline changes” and “C4d positive staining without evidence of rejection” were not considered as rejection in our study (23).

### Data processing and statistical analysis

All the raw data were exported from the STCS database and the subsequently processed with R (version 4.0.3) and RStudio (version 1.3.1093) using the packages “dplyr” (1.0.7), “ggplot2” (3.3.6), “lubridate” (1.8.0), “pacman” (0.5.1), “rio” (0.5.29), “stats” (4.0.3), “survminer” (0.4.9), tibble” (3.1.6) and “tidyr” (1.1.4). Kaplan-Meier analysis was the method used to present the “time-to-event” data, such as the incidence of the ABMR, TCMR, graft survival and patient survival. Statistical significance was calculated with a log-rank test to compare the Kaplan-Meier survival analysis between groups. One-way analysis of variance (ANOVA) followed by Tukey’s multiple comparisons as a *post hoc* test was used to analyze the distribution of DSA MFI in different groups. For all the tests, p<0.05 was considered to indicate the statistical significance.

## Results

### Study population characteristics

An overview of the included patients is shown in **Figure 1A**. After exclusion of pediatric transplants as well as patients with incomplete baseline data or who refused consent a total of 952 LD transplantations performed in Switzerland between 2008 and 2017 were included in the final analysis. Within this subgroup, 149 transplants were performed in the ABOi setting whereas 803 were ABOc. In the ABOc setting 14.6% were performed in the presence of a pre-transplant DSA as compared to 10.7% DSA positive transplant in the ABOi group. Follow-up times did not differ markedly between ABOc and ABOi patients (**Figure 1B**). The majority of DSA positive transplants were performed in the setting of low MFI DSA (<5000) in both ABOc and ABOi patients (**Figure 1C**).

### ABOi transplants show comparable transplant outcome

**Figure 2:**
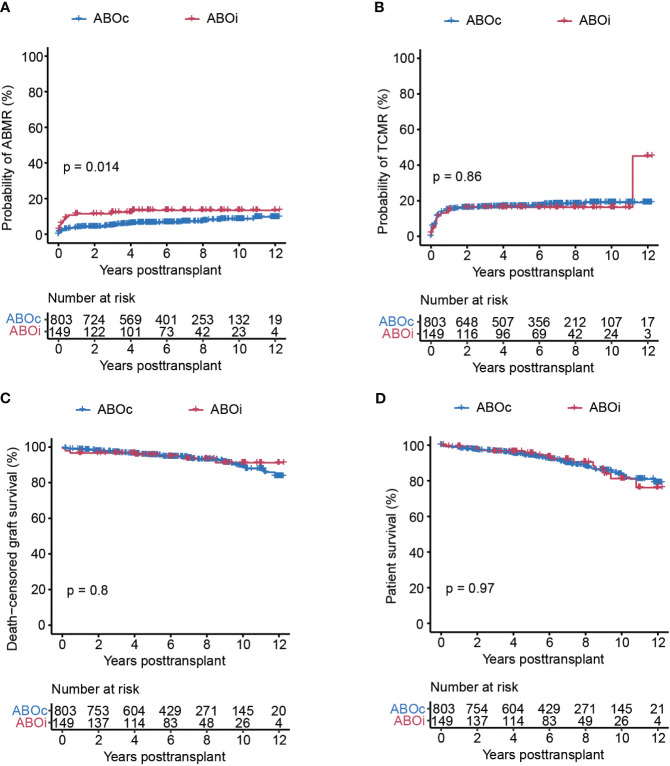
ABOi patients showed significantly higher risk for the development of ABMR as compared to ABOc patients, but similar outcomes regarding TCMR, graft survival and overall survival. Cumulative incidence of ABMR **(A)**, TCMR **(B)**, death-censored graft survival **(C)**, and overall patient survival **(D)** in the ABOc and ABOi patients respectively. Log-rank test was used to test p value of the Kaplan-Meier survival curves for **(A–D)**.

We compared transplant outcome in terms of ABMR, TCMR, death-censored graft survival as well as overall patient survival between ABOi and ABOc patients. We could detect a small but significant increase of ABMR in ABOi transplants, which appeared to be caused primarily by an increased risk in this group for ABMR development during the first year after transplantation (**Figure 2A**). We could not detect a similar incased risk for the development of TCMR where the risk was comparable between both ABOi and ABOc transplants (**Figure 2B**). The detected small increased risk of ABMR did not however translate to an inferior graft survival in ABOi transplants who in our cohort showed excellent long-term graft survival that was very similar to ABOc transplants (**Figure 2C** and **Table 2**). In contrast to some previous studies, ABOi was in our study not associated with a decreased overall survival (**Figure 2D**) (8, 9). In summary, we show similar excellent transplant outcome in recipients of a LD ABOi kidney as compared to recipients of an ABOc kidney.

**Table 2:** Clinical outcomes post-transplantation in ABO compatible and ABO incompatible kidney transplants.

Outcome	ABO-compatible (n=803)	ABO-incompatible (n=149)	P-value
ABMR, %	53 (6.6%)	18 (12.1%)	<0.0001
TCMR, %	129 (16.1%)	23 (15.4%)	0.699
Graft survival, %	749 (93.3%)	140 (94.0%)	0.535
Patient survival, %	721 (89.8%)	134 (89.9%)	0.915

### Pre-transplant DSA are associated with inferior transplant outcome regardless of ABO compatibility status

**Figure 3:**
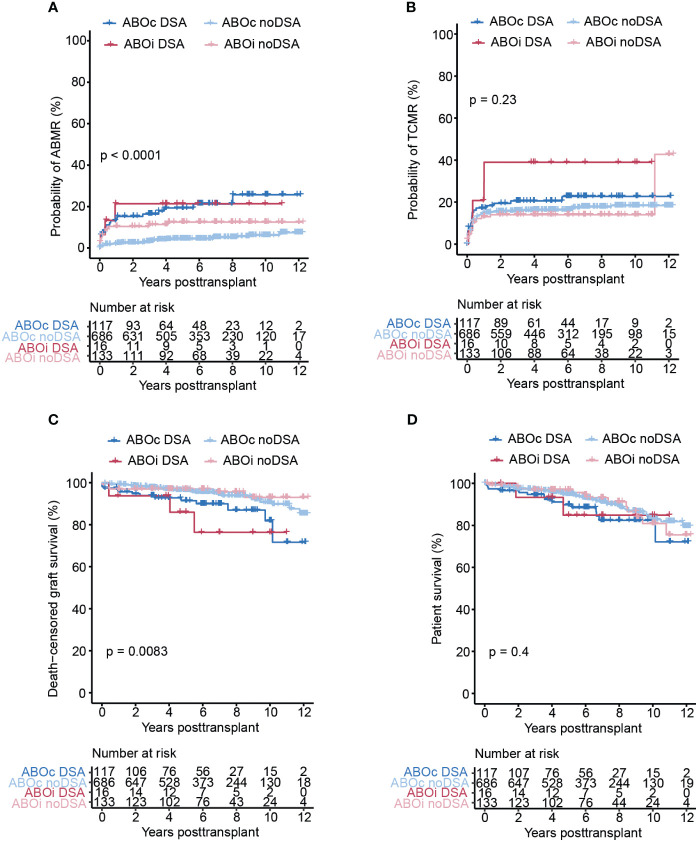
Pre-transplant DSA are associated with significantly increased risk of ABMR and graft loss in both ABOc and ABOi transplants. Cumulative incidence of ABMR **(A)**, TCMR **(B)**, death-censored graft survival **(C)**, and overall patient survival **(D)** in the ABOc and ABOi patients respectively stratified on DSA status. Log-rank test was used to test p value of the Kaplan-Meier survival curves for **(A–D)**.

We next sought to investigate the impact of pre-transplant DSA in both the ABOi and ABOc LD kidney transplant setting. DSA were coupled to significantly increased risk for the development of ABMR in both the ABOc and ABOi setting, and there was no evidence of a further increased ABMR risk within our cohort in ABOi DSA patients (**Figure 3A**). With regards to TCMR we found a trend for a higher risk in DSA positive ABOi patients but this did not reach significance (**Figure 3B**). Both ABOi and ABOc DSA positive patients showed significantly increased graft loss as compared to DSA negative ABOi and ABOc patients (**Figure 3C**). We could not detected a marked difference in graft survival between DSA positive patients based on ABO compatibility. Overall survival was similar for all our investigated subgroups with a slight trends towards worse survival in DSA positive patients at 4-6 years post transplantation (**Figure 3D**). In summary, DSA positivity was associated with inferior transplant outcome regardless of ABO compatibility status and we found no evidence of an additive effect of ABOi and DSA positivity in our study.

### DSA HLA class target and MFI

**Figure 4:**
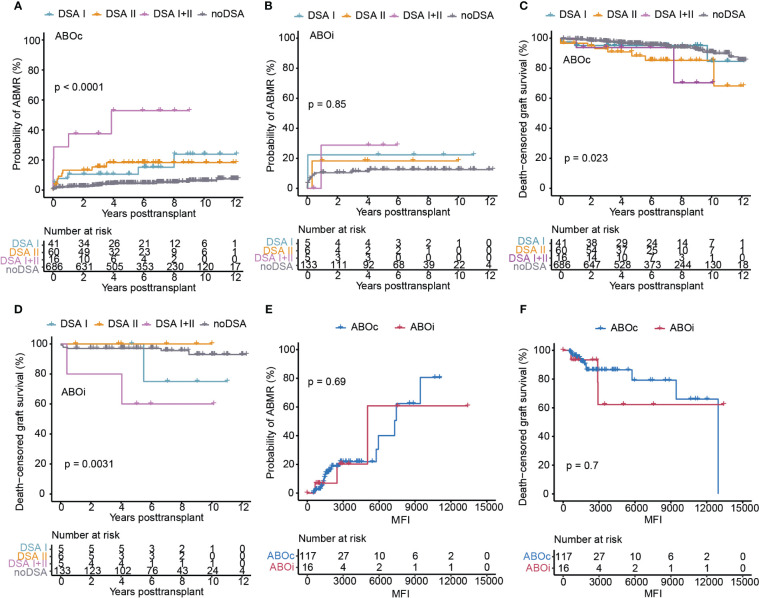
The influence of HLA-DSA Class and DSA MFI on the transplantation outcomes in ABOc and ABOi DSA positive transplants. Cumulative incidence of ABMR in ABOc **(A)** and ABOi **(B)** transplants, stratified on DSA Class or in patients without DSA (noDSA). Death-censored graft survival in ABOc **(C)** and ABOi **(D)** transplants stratified on DSA Class or in patients without DSA (noDSA). Cumulative incidence of ABMR **(E)** and death-censored graft survival **(F)** in the ABOc and ABOi DSA positive transplants in relation to cumulative DSA MFI value. Log-rank test was used to test p value of the Kaplan-Meier survival curves for **(A–F)**. DSA I, DSA directed at HLA Class I; DSA II, DSA directed at HLA Class II; DSA I+II, DSA directed at a combination of HLA Class I and II.

We next sought to investigate the impact of the DSA target locus (HLA Class I or II) as well as MFI on transplant outcome in the ABOc and ABOi setting. In ABOc transplants, the highest probability of developing ABMR was seen in the group with multiple DSA targeting both HLA Class I and II antigens whereas the risk appeared to be similar in the setting of only Class I or Class II targeting DSA (**Figure 4A**). For ABOi transplants, a similar picture was visible with the highest risk again being associated with combined DSA I and II, even though this did not reach significance likely due to the small number of patients in each subgroup (**Figure 4B**). Graft survival was significantly worse for both transplants performed in the setting of DSA II or DSA I + II in ABOc recipients whereas Class I directed DSA did not appear to significantly impact graft survival (**Figure 4C**). For ABOi recipients we could again detect a significantly elevated risk of graft loss in patients with a combination of Class I and Class II DSA (**Figure 4D**). For patients with isolated Class I or Class II DSA the picture was less clear and we did not observe any graft loss event within the ABOi DSA positive group in the setting of isolated Class II DSA (**Figure 4D**). In DSA positive transplants the MFI of the detected DSA also had a large effect on the risk of ABMR development and graft loss (**Figures 4E, F**). There was however no demonstrable differences between DSA positive ABOc and ABOi patients with regards to the influence of MFI (**Figures 4E, F**). In summary, a combination of Class I and Class II directed DSA as well as high MFI DSA were in our study associated with inferior outcomes regardless of ABO compatibility.

## Discussion

The pre-transplant immunological risk assessment in the setting of LD kidney transplantation is primarily based on estimating the risk of the immunogentical mismatch between the recipient and donor as well as evaluating possible evidence of pre-existing donor specific alloimmunity. With sometimes multiple alternative options for transplantation available such as additional living donors, entering into a kidney paired donation (KPD) program or opting to wait for a better immunologically matched deceased donor the decisions surrounding the acceptance or decline of a possible LD based on immunological grounds can be challenging. The immunological risk must also be appreciated in the context of other relevant factors such as metabolic and age mismatch. ABO blood group incompatibility is also a pre-transplant immunological risk factor that influences the immunosuppressive therapy and can affect transplant outcome. Therefore, some transplant centers prefer to perform an ABOc transplant in the setting of a KPD program instead of a regular directed ABOi LD transplantation. Our data from the STCS would argue that ABOi transplants do not show a worse outcome as compared to ABOc transplants within the Swiss Transplant program. This is also in line with previous studies that have not shown an additive risk of graft loss in the combined ABOi and DSA positive setting as compared to DSA positive transplant in the ABOc setting (8, 20). We did detect a significant increase in ABMR risk associated with ABOi, which occurred mainly during the first year post-transplantation, but this did not appear to translate into increased graft loss or decreased patient survival. This circumstance may also arise from the Banff classification, wherein the ubiquitous occurrence of C4d in the ABOi setting, coupled with the presence of anti-blood group antibodies, inherently satisfies two criteria indicative of active antibody-mediated rejection (ABMR). Consequently, only minimal additional biopsy anomalies also without evidence for microvascular inflammation would be necessary for an ABMR diagnosis. It is crucial to note, however, that such diagnoses may not genuinely reflect ongoing and clinically significant ABMR (24, 25). Our data are in line with previous studies showing similar graft and patient survival in ABOc and ABOi transplants (3, 4). They are however not consistent with other studies that show either increased graft loss or reduced patient survival associated with ABOi (8–10). These differences may in part be related to differences in transplant protocols concerning induction therapy as well as maintenance immunosuppression used in the setting of ABOi (26). With the increased risk of ABMR observed in our study and in previous reports it could be reasonable to postulate that the combination of ABOi and DSA would work as additive risk factors. This idea could lead to a policy of reluctance in performing DSA positive ABOi transplants, which can also to some extent be visualized in our data (14.6% DSA positive ABOc transplants compared to 10.7% DSA positive ABOi transplants), even though there is no conclusive data to support this strategy. We were able in our study to show a significant negative effect of pre-transplant DSA on graft outcome for both in ABOc and ABOi transplants but we could not observe a clear signal for an additive risk in the ABOi setting. We did observe a trend towards increased risk of TCMR in the ABOi DSA positive setting even though this did not reach statistical significance and we could not show an increased graft loss in this group as compared to DSA positive ABOc transplants within the observation time of our study. Our findings are interesting and could have a direct impact on decisions made in the setting of ABOi LD kidney transplantations. A possible reason for the similar outcome in DSA positive patients regardless of ABO blood group incompatibility could be that the B cell depleting induction therapy, used in all of the transplanted ABOi patients in our study, may somewhat offset a possible additive effect of ABOi and DSA. Interestingly a recent study has indicated that the risk of DSA development in ABOi transplants might be reduced as compared to ABOc (27).

Our study has several limitations related to the multicenter design and long inclusion period, including differences in induction and maintenance immunosuppressive therapies at the different centers, as well as related to evaluation of SAB results and individual procedures for the diagnosis and therapy of rejection. The number of DSA positive ABOi transplantations captured in our study is also small (n=16) and our data should therefore be interpreted with caution and needs to be confirmed in a larger cohort. Development of *de novo* DSA or antibody kinetics of pre-transplant DSA post transplantation is not captured within the STCS database and we are therefore unable to assess the effect of these important markers on the outcome of transplantation.

In summary, we present long-term data on the effect of pre-transplant DSA in the setting of ABOi LD kidney transplantation. Our study is the first to investigate the long-term effects of DSA in the ABOi setting with a complete virtual crossmatch and argues that pre-transplant DSA risk could perhaps be evaluated similarly regardless of ABO compatibility status.

## Data availability statement

The original contributions presented in the study are included in the article/supplementary materials, further inquiries can be directed to the corresponding author/s.

## Ethics statement

The studies involving humans were approved by Cantonal ethics committee of Zurich (BASECNr. 2021-0083). The studies were conducted in accordance with the local legislation and institutional requirements. The participants provided their written informed consent to participate in this study.

## Author contributions

YD: Writing – original draft, Writing – review & editing. LF: Writing – review & editing. CW: Writing – review & editing. OdR: Writing – review & editing. JV: Writing – review & editing. SF-L: Writing – review & editing. DG: Writing – review & editing. MG: Writing – review & editing. IB: Writing – review & editing. UW: Writing – review & editing. DS: Writing – review & editing. TS: Writing – review & editing. SS: Writing – review & editing. JN: Writing – original draft, Writing – review & editing.

## Group member of The Swiss Transplant Cohort Study

The members of the Swiss Transplant Cohort Study are: Patrizia Amico, Adrian Bachofner, Vanessa Banz, Sonja Beckmann, Guido Beldi, Christoph Berger, Ekaterine Berishvili, Annalisa Berzigotti, Pierre-Yves Bochud, Sanda Branca, Heiner Bucher, Anne Cairoli, Emmanuelle Catana, Yves Chalandon, Sabina De Geest, Sophie De Seigneux, Michael Dickenmann, Joëlle Lynn Dreifuss, Michel Duchosal, Thomas Fehr, Sylvie Ferrari-Lacraz, Jaromil Frossard, Christian Garzoni, Déla Golshayan, Nicolas Goossens, Fadi Haidar, Jörg Halter, Dominik Heim, Christoph Hess, Sven Hillinger, Hans Hirsch, Patricia Hirt, Linard Hoessly, Günther Hofbauer, Uyen Huynh-Do, Franz Immer, Michael Koller, Andreas Kremer, Thorsten Krueger, Christian Kuhn, Bettina Laesser, Frédéric Lamoth, Roger Lehmann, Alexander Leichtle, Oriol Manuel, Hans-Peter Marti, Michele Martinelli, Valérie McLin, Katell Mellac, Aurélia Merçay, Karin Mettler, Nicolas Müller, Ulrike Müller-Arndt, Beat Müllhaupt, Mirjam Nägeli, Graziano Oldani, Manuel Pascual, Jakob Passweg, Rosemarie Pazeller, Klara Posfay-Barbe, David Reineke, Juliane Rick, Anne Rosselet, Simona Rossi, Rössler, Silvia Rothlin, Frank Ruschitzka, Thomas Schachtner, Stefan Schaub, Alexandra Scherrer, Dominik Schneidawind, Aurelia Schnyder, Macé Schuurmans, Simon Schwab, Thierry Sengstag, Federico Simonetta, Jürg Steiger, Guido Stirniman, Ueli Stürzinger, Christian Van Delden, Jean-Pierre Venetz, Jean Villard, Julien Vionnet, Madeleine Wick, Markus Wilhlem, Patrick Yerly.
